# Growth factors expression and ultrastructural morphology after application of low-level laser and natural latex protein on a sciatic nerve crush-type injury

**DOI:** 10.1371/journal.pone.0210211

**Published:** 2019-01-09

**Authors:** Fernando José Dias, Valéria Paula Sassoli Fazan, Diego Pulzatto Cury, Sonia Regina Yokomizo de Almeida, Eduardo Borie, Ramón Fuentes, Joaquim Coutinho-Netto, Ii-sei Watanabe

**Affiliations:** 1 Department of Integral Dentistry, CICO—Research Centre in Dental Sciences, Dental School, Universidad de La Frontera, Temuco, Chile; 2 Department of Surgery and Anatomy, School of Medicine of Ribeirao Preto, University of Sao Paulo, Ribeirao Preto, Brazil; 3 Department of Anatomy, Institute of Biomedical Sciences, University of Sao Paulo, Sao Paulo, Brazil; 4 Department of Pharmacology, School of Medicine of Ribeirao Preto, University of Sao Paulo, Ribeirao Preto, Brazil; Massachusetts General Hospital, UNITED STATES

## Abstract

The effects of low-level laser therapy (LLLT) and natural latex protein (F1, *Hevea brasiliensis*) were evaluated on crush-type injuries (15kg) to the sciatic nerve in the expressions of nerve growth factor (NGF) and vascular endothelium growth factor (VEGF) and ultrastructural morphology to associate with previous morphometric data using the same protocol of injury and treatment. Thirty-six male rats were allocated into six experimental groups (n = 6): 1-Control; 2-Exposed nerve; 3-Injured nerve; 4-LLLT (15J/cm^2^, 780nm, 30mW, Continuous Wave) treated injured nerve; 5-F1 (0,1mg) treated injured nerve; and 6-LLLT&F1 treated injured nerve. Four or eight weeks after, sciatic nerve samples were processed for analysis. NGF expression were higher (p<0.05) four weeks after in all injured groups in comparison to Control (Med:0.8; Q1:0; Q3:55.5%area). Among them, the Injured (Med:70.7; Q1:64.4; Q3:77.5%area) showed the highest expression, and F1 (Med:17.3; Q1:14.1; Q3:21.7%area) had the lowest. At week 8, NGF expressions decreased in the injured groups. VEGF was expressed in all groups; its higher expression was observed in the injured groups 4 weeks after (Injured. Med:29.5; F1. Med:17.7 and LLLT&F1. Med:19.4%area). At week 8, a general reduction of VEGF expression was noted, remaining higher in F1 (Med:35.1; Q1.30.6; Q3.39.6%area) and LLLT&F1 (Med:18.5; Q1:16; Q3:25%area). Ultrastructural morphology revealed improvements in the treated groups; 4 weeks after, the F1 group presented greater quantity and diameter of the nerve fibers uniformly distributed. Eight weeks after, the F1 and LLLT&F1 showed similar characteristics to the non-injured groups. In summary, these results and our previous studies indicated that F1 and LLLT may favorably influence the healing of nerve crush injury. Four weeks after nerve injury F1 group showed the best results suggesting recovery acceleration; at 8th week F1 and LLLT&F1 groups presented better features and higher vascularization that could be associated with VEGF maintenance.

## Introduction

Nerves constitute the functional part of the peripheral nervous system (PNS) formed by fascicles of myelinated and unmyelinated nerve fibers, composed of motor, sensory and autonomic portions [1, 2]. Approximately 100,000 patients are submitted to surgery annually to recover peripheral nerves in the U.S.A. and Europe. These injuries have a strong negative impact on the quality of life of the population, resulting in paralysis, anesthesia, lack of control and painful neuropathy. Representing a major cause for morbidity and disability, and pose substantial costs for society from a global perspective. Although peripheral nerve fibers have considerable potential for regeneration, spontaneous recovery is generally poor and may result in sequelae, especially in the case of large nerve defects [1, 3–7].

The treatment of peripheral nerve lesions still is a challenge. Despite advances in understanding of nervous regeneration in microsurgical techniques, functional recovery remains unsatisfactory [4, 6–10]. Thus, there is an increasing need for research that achieves innovative treatments for regeneration of peripheral nerves using an interdisciplinary approach [1]. Stimulation systems, biological stimuli and physical stimuli have shown encouraging results in the regeneration of peripheral nerves [3].

There is interest in the potential therapeutic value of low-level laser therapy (LLLT), for treatment of central nervous system and PNS injury and disorders, such as strokes, spinal cord injury, traumatic brain injury, multiple sclerosis, or Parkinson’s disease, which cause functional impairments [7, 11]. The photochemical and photobiological effects produced by LLLT at the cellular level may induce trophic conditions, such as neurite outgrowth, metabolic production, secretion of neural factors, and inhibition of the inflammatory process that are necessary for nerve regeneration [12, 13].

Specifically, LLLT (904 nm, 26.3 mW and 4 J/cm^2^) applied on the sciatic nerve of rats submitted to neuropraxia revealed an increase in the number of neurons, Schwann cells and large myelinated axons [14]. On the sectioned sciatic nerve (neurotmesis), LLLT irradiation (808 nm, 30 mW and 50 J/cm^2^) resulted in higher nerve fiber density. The axonal diameters of these fibers were higher in the irradiated animals (660 nm, 30 mW and 50 J/cm^2^), suggesting functional gait recovery associated with improved morphometric parameters [15]. In an acute sciatic nerve crush lesion, the application of LLLT (660 nm, 40 mW and 60 J/cm^2^) increased the activities of MMP-9 and TNF-α protein in nerve injury modulating inflammation [16].

In the 1990s, a new biocompatible material derived from the tree *Hevea brasiliensis* (rubber tree) was developed at the Laboratory of Neurochemistry of the Medical School of Ribeirão Preto—University of São Paulo, the F1 protein [17]. Initial studies demonstrated promising results using the F1 protein. It was reported that the stimulation of angiogenesis, cell adhesion and formation of extracellular matrix, leads to the acceleration of wound healing in cutaneous tissue, pericardium, esophagus, abdominal wall, tympanum and blood vessels with no evidence of hypersensitivity [18, 19].

On a sciatic nerve injury (neurotmesis), a neurotube made of latex, and containing the F1 protein improved the quality of nerve regeneration, nerve impulse conduction and gait [20]. Our previous studies have also shown improvement in nerve regeneration using F1 protein carried by a hyaluronic acid hydrogel [21–24].

Growth factors or trophic factors are polypeptides that bind to specific cell membrane surface receptors to initiate signaling pathways that regulate proliferation, survival, migration and differentiation. These neurotrophic factors constitute a class of trophic factors that act on cells of the peripheral and central nervous systems. Neurotrophic factors, including the members of the neurotrophin family (NGF, BDNF, NT3 and NT4) play an important role in the regulation of growth, survival, and differentiation of neurons in the central and peripheral nervous systems. Since the 1950s, these factors have been studied in traumatic injury models [25–29].

The family of vascular endothelial growth factors (VEGFs) stimulate the growth of new blood vessels, and are associated with the lesions of the nervous tissue [30, 31]. The VEGFA is the most studied family member, being a critical regulator of angiogenesis that is usually referred as VEGF [32]. Nerve growth factor (NGF) is a neurotrophic factor from the neurotrophin family, known to specifically act on small primary sensory and sympathetic neurons [33, 34]. NGF promotes the proliferation, survival, protection and differentiation of neurons and oligodendrocytes. In addition, NGF modulates the repair of injured axons and regulates the key structures of the proteins that constitute the myelin [35–37].

The aim of this study was to contribute to the understanding of the recovery of peripheral nerve lesions by evaluating the effect of the LLLT associated with the F1 protein on sciatic nerve axonotmesis of rats. The expression of NGF and VEGF is analyzed through immunohistochemical reactions and its association with ultrastructural morphology.

## Materials and methods

Thirty-six male Wistar rats (2 months old, 200–250 g) were allocated into six experimental groups (n = 6). The animals were kept in a bioterium in polypropylene boxes with up to four animals per box, at controlled temperature (22 to 24 ^o^C), 12 hours of daily illumination and air changes, with food and water "*ad libitum*". The study protocol was approved by the Universidad de La Frontera Ethics Committee on Animal Use under protocol no. 125/16, on March of 2017, following the norms and international laws of animal experimentation.

### Nerve injury

The animals were anesthetized with ketamine and xylazine (75/10 mg/kg), and submitted to tricotomy on the lateral side of the left pelvic leg (hind paw). The incision site was standardized by the position of two bone processes of the iliac crest, the superior and inferior ventral spines. A small incision (~2 cm) was made on the skin perpendicular to the union line of these bone processes. The incision was performed by receding approximately 2 cm in the caudal direction on the lateral face of the pelvic limbs of the animal. Divulsion of the superficial gluteus maximus and femoral biceps muscles was then performed exposing the sciatic nerve. The animals were then placed in a nerve injury apparatus made specifically for this purpose. The load applied to the sciatic nerve was constant at 15 kg for 10 minutes, in a circular crushing area (~0.28 cm^2^, 5.2 MPa). After injury, nerves were repositioned, the skin was sutured with 4–0 nylon, and the animals received 0.2 ml/kg of anti-inflammatory (Banamine-Schering Plow, Flunixina meglumine 10 mg/ml) and 0.3 ml/kg of broad-spectrum anti-inflammatory (pentabiotic—Fort Dodge) [38].

### F1 protein purification and application

For the latex protein purification, ammonium latex was diluted in 2.2% acetic acid. The diluted latex was homogenized and left at room temperature for 30 min. Latex serum was separated from the rubber and submitted to chromatographic separation using ionic exchange chromatography with DEAE-celluloses. Serum was diluted in distilled water and the pH was adjusted to 9.0. This material was applied to the chromatographic column at room temperature and eluted with 0.01 M ammonium bicarbonate in a growing gradient of NaCl (0.15, 0.25 and 1.5 M). The material was collected under a flux of 7 ml/min and monitored for absorbance at 280 nm. Peak 1 fraction (F1) was then submitted to distilled water dialysis, lyophilisation and stored at -20°C [39].

The F1 protein was associated to a hyaluronic acid hydrogel that was used as a carrier [40]. Hyaluronic acid (Nikko Chemicals, Nikkol Group, Japan), isolated from gram-negative bacteria, was used at the final concentration of 1% (10mg/ml), and F1 was used at a concentration of 0.1% (1mg/ml). Both were mixed and filtered in a sterile system (0.22 μm Millipore filters) and stored in sterile culture flasks. After injury and prior to suturing [21–23], the mixture was applied on the nerve with the aid of a micropipette (100 μl).

### Low-level laser irradiation

The "Twin Laser—Mm Optics" (Ga-Al-As) apparatus was used, with an application area of 0.04 cm^2^ (spot). Six irradiation sessions were performed on alternate days at three points on the region of the injured nerve, corresponding to the proximal, distal regions and the area of the lesion itself. Irradiation parameters in the postoperative phase are presented in Table 1.

**Table 1:** Low-level laser irradiation parameters.

**Power**	30 mW
**Intensity**	0.75 W/cm^2^
**Energy Density**	15 J/cm^2^
**Wavelength**	780 nm
**Application time (per point)**	20 s
**Number of application points**	3
**Wave Type**	Continuous Wave (CW)
**Beam Direction**	Perpendicular to the Tissue
**Energy deposited (per point)**	0.6 J
**Application spot area**	0.04cm^2^
**Number of sessions**	6 (Alternate Days)

### Experimental groups

The study groups were evaluated 4 and 8 weeks after of the nerve injury. The protocols of the experimental groups are described below:

- **Control group (Control)**—Animals anesthetized and kept in lateral decubitus for 10 minutes, simulating the stress of the intervention.
- **Exposed group (Exposed)**—Animals anesthetized, sciatic nerve exposed and positioned on the support of the nerve lesion apparatus for 10 minutes [38].
- **Injury group (Injury)**—Anesthetized animals, sciatic nerve submitted to crush-type injury.
- **Low-level laser group (LLLT)—**Anesthetized animals, sciatic nerve submitted to crush-type injury. Animals submitted to the LLLT irradiation protocol.
- **Latex F1 Protein group (F1)—**Anesthetized animals, sciatic nerve submitted to crush-type injury. F1 protein applied at the lesion site.
- **Low-level laser and Latex F1 Protein group (LLLT&F1)**—Anesthetized animals, sciatic nerve submitted to crush-type injury. F1 protein was applied at the lesion site and animals subjected to the LLLT irradiation protocol.

### Immunohistochemical reaction—anti-NGF and anti-VEGF

The animals were euthanized with anesthetic overdose (ketamine and xylazine 150 and 10 mg/kg, respectively). The obtained nerve samples were placed in a plastic canister containing Optimal cutting temperature compound and frozen in isopentane cooled by liquid nitrogen (-196°C). The nerve samples were transferred to the cryostat microtome chamber (-20°C), and cross-sections of 10 μm thickness were mounted on histological adhesive slides.

Immunostaining was performed using the primary antibodies: anti-NGF (Abcam-ab6198, USA), anti-VEGFA (SantaCruz-sc152, USA). Secondary antibody (polymer "histofine HRP Nichirei", 414341F, Japan) and diaminobenzidine (Spring, DAB-125, USA). The histological sections were covered with H_2_O_2_ (3% - 10 vol.) for subsequent blocking of the endogenous peroxidase for 10 minutes and non-specific binding with Protein Block—Spring (DPB-125) for 1 h. Samples were rinsed in PBS, and then incubated with the primary antibodies (anti-NGF, concentration 1: 400 and anti-VEGF, concentration 1: 100) overnight at 4°C. The next day, the sections were washed in PBS and incubated for 2 h with the secondary antibody HRP (room temperature). The reaction was revealed with diaminobenzidine solution (0.5 mg/ml) and liquid hydrogen peroxide (0.005 ml/100 ml) in PBS for 1 minute. The sections were rinsed in PBS, counterstained with hematoxylin, washed with distilled water and mounted with Entellan [41].

### Ultrastrutural analysis–transmission electron microscopy

For ultrastructural analysis, nerve samples were fixed in 2.5% glutaraldehyde for 2 h, post-fixed in OsO_4_ 1% at 4°C for 2 h, dehydrated in an ethanol series and propylene oxide, and embedded in Spurr resin. Finally, ultrathin sections (60 nm) were mounted on 200 mesh grids, counterstained with uranyl acetate and lead citrate. Sections were then examined under a JEOL 1010 electron microscope operating at 80 kV at the Institute of Biomedical Sciences, University of Sao Paulo.

### Semi-quantitative analysis—NGF and VEGF

**Fig 1:**
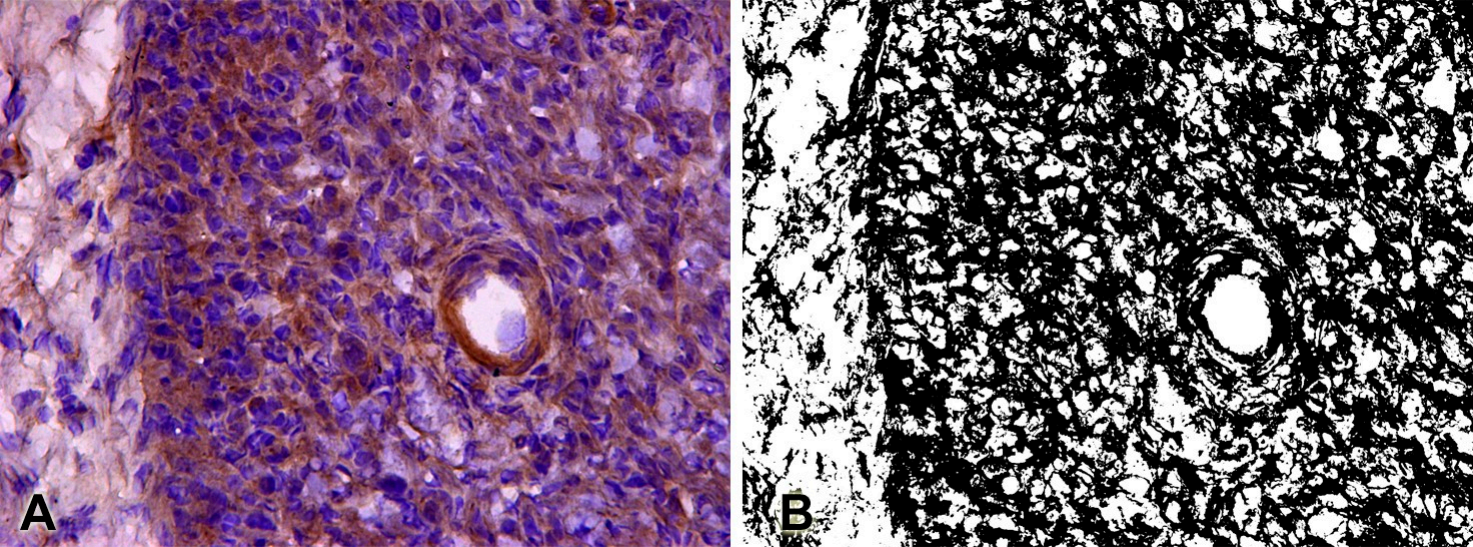
Semi-quantitative analysis of immunostaining. ***A*.** Photomicrography of NGF immunolabeling, presented in brown. ***B*.** Photomicrography transformed to binary (black and white) by the "Color threshold" feature of the ImageJ software. These binary images were used for counting pixels for the semi-quantitative analysis of growth factors expressions.

Semi-quantitative analysis of the immunoreactivity of the NGF and VEGF factors was performed by quantifying the pixels of the binary images that provided the positively labeled area density (%). In order to perform this area measurement, ImageJ software (NIH, USA) was used, using the "Color threshold" (B & W) feature to select which color range should be considered black and all other colors considered white (Fig 1).

### Statistical analysis

Statistical analysis was performed using the SigmaPlot 12.0 software (Systat software Inc, San Jose, CA, USA). The Shapiro-Wilk test was selected to assess data normality. Data from the semi-quantitative immunoreactivity analysis of NGF and VEGF did not show normal distribution. Thus, we used the Kruskal-Wallis followed by a Dunn’s post-hoc test. Data are presented as the median (Q2, 50%) as central tendency measure, and the data dispersion as Q1 (25%), and Q3 (75%).

## Results

### Growth factors expression–immunohistochemical analysis

#### NGF–nerve growth factor

**Fig 2:**
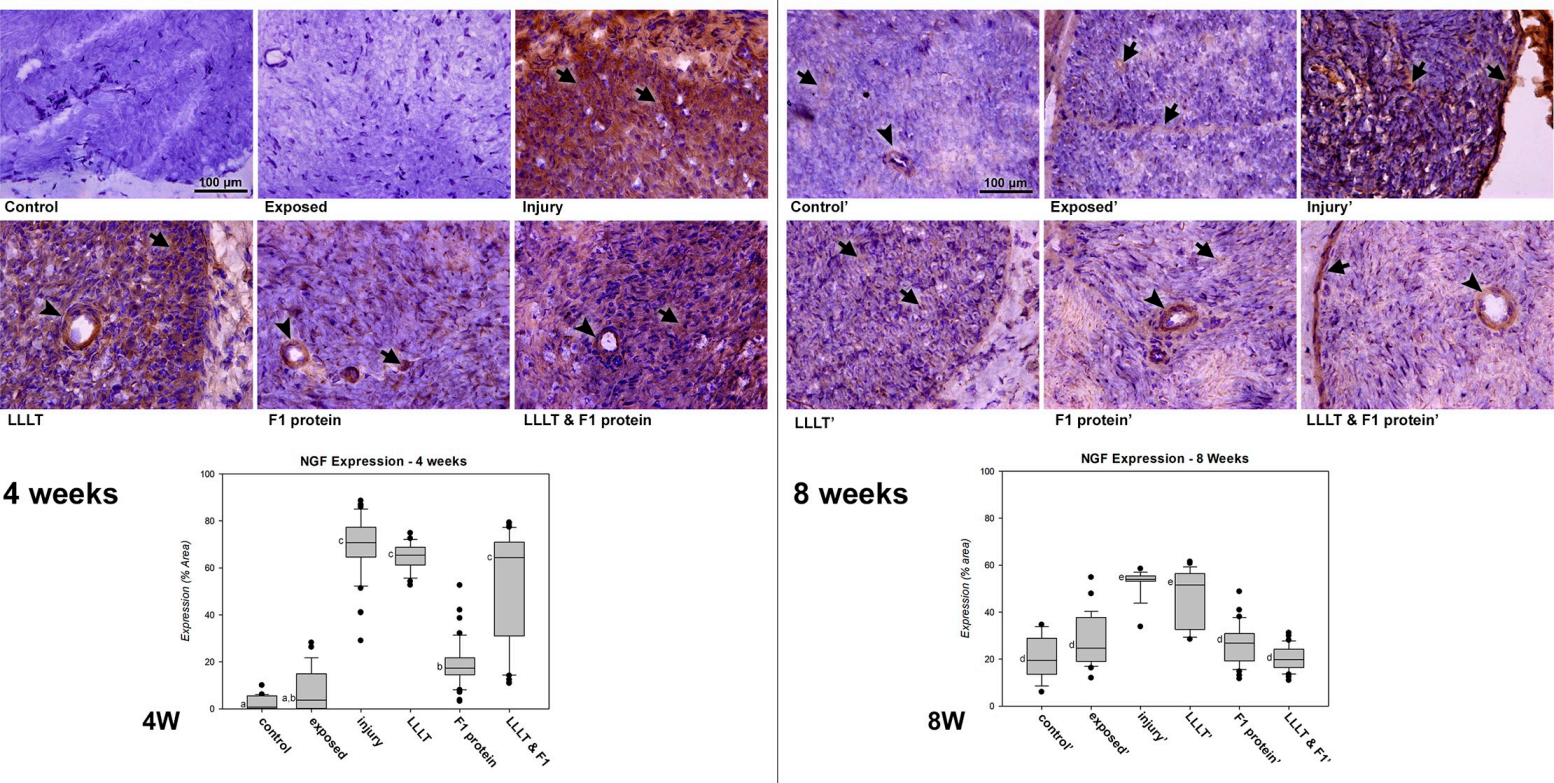
Immunoreactivity and semi-quantitative analysis of NGF after nerve injury. NGF expression (brown, arrow; arrowhead—endothelial cells) 4 weeks after nerve injury in the groups: ***Control*.** Control group; ***Exposed*.** Exposed nerve without injury; ***Injury*.** Injured nerve without treatment; ***LLLT*.** Injured nerve treated with LLLT; ***F1 protein*.** Injured nerve treated with F1 protein; and ***LLLT & F1 protein*.** Injured nerve treated with LLLT and F1 protein (Mags: X400). ***4W*.** NGF expressions graph of the period, same letters (a, b and c) represent equalities among the groups (p > 0.05). NGF expression (brown, arrow; arrowhead—endothelial cells) 8 weeks after nerve injury in the groups: ***Control’*.** Control group; ***Exposed’*.** Exposed nerve; ***Injury’*.** Injured nerve; ***LLLT’*.** Injured nerve with LLLT; ***F1 protein’***. Injured nerve with F1 protein; and ***LLLT & F1 protein’*.** Injured nerve with LLLT and F1 protein (Mags: X400). ***8W*.** NGF expressions graph of the period, the same letters (d and e) represent equalities among the groups (p > 0.05).

Four weeks after the nerve injury (Fig 2), the study groups Control and Exposed were similar, presenting the lowest immunoreactivity for NGF. Among the injured groups, Injury, LLLT and LLLT&F1 were similar and showed significantly higher immunoreactivity (p < 0.05) compared to non-injured groups, immunostaining was observed mainly among the nerve fibers and glial cells; however, it was possible to observe endothelial cell wall marking. The F1 protein group was the only group submitted to nerve injury that presented lower immunoreactivity compared to the other injured groups (Injury, LLLT and LLLT&F1) in this period of analysis, with similar values of the Exposed group (p > 0.05). Immunolabeling was observed in the F1 protein group with less intensity among nerve fibers, glial cells and also observed in the endothelial cells.

Eight weeks after nerve injury (Fig 2), the NGF immunoreactivity of all injured groups was reduced in relation to the previous period of analysis. The Control, Exposed, F1 and LLLT&F1 groups showed similar immunoreactivity (p > 0.05). Immunostaining in these groups was observed mainly in the perineurium, epineurium and endothelial cells. Injury and LLLT groups presented the highest immunoreactivity (p < 0.05) in comparison to the other groups. This is because immunostaining of these groups still showed considerable concentration between nerve fibers and glial cells.

The expression of NGF in groups with the same experimental methodology in the different periods (4 and 8 weeks) revealed significant differences in all analyzed pairs (Groups Control, Exposed, Injury, LLLT and LLLT&F1 with p <0.001 and F1 with p = 0.002). Groups Control, Exposed and F1 showed increased NGF expression, whereas groups Injury, LLLT and LLLT&F1 groups showed a reduction 8 weeks after of injury.

#### VEGF—vascular endothelial growth factor

**Fig 3:**
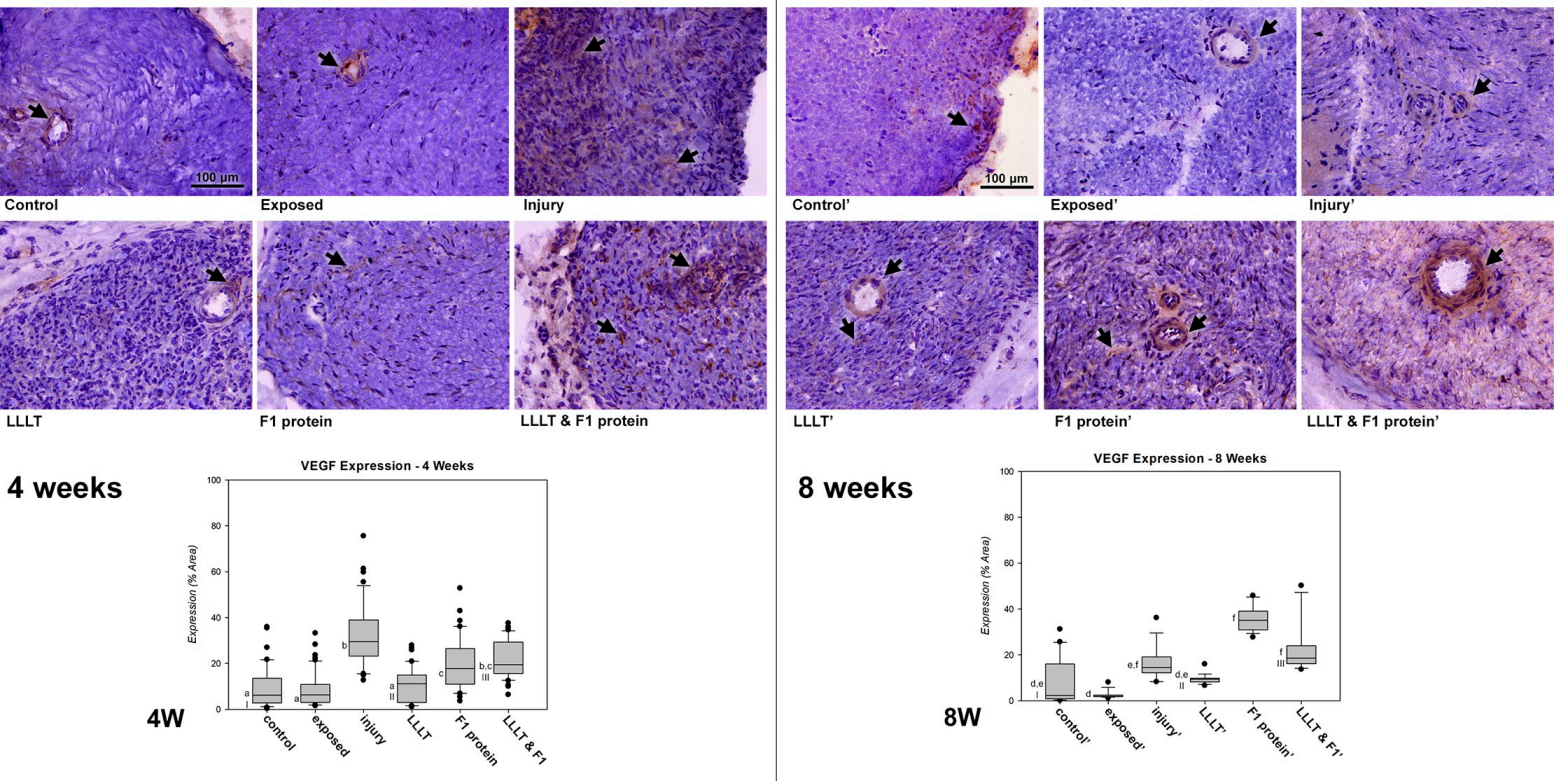
Immunoreactivity and semi-quantitative analysis of VEGF after nerve injury. VEGF expression (brown, arrow) **4 weeks** after nerve injury in the groups: ***Control*.** Control group; ***Exposed*.** Exposed nerve without injury; ***Injury*.** Injured nerve without treatment; ***LLLT*.** Injured nerve treated with LLLT; **F1 protein.** Injured nerve treated with F1 protein; and ***LLLT & F1 protein*.** Injured nerve treated with LLLT and F1 protein (Mags: X400). **4W.** Graph of VEGF expressions during the period, same letters (a, b and c) represent equalities among the groups (p > 0.05). VEGF expression (brown, arrow) **8 weeks** after nerve injury in the groups: ***Control’***. Control group; ***Exposed’***. Exposed nerve; ***Injury’***. Injured nerve; ***LLLT’***. Injured nerve with LLLT; **F1 protein’**. Injured nerve with F1; and ***LLLT & F1 protein’***. Injured nerve with LLLT and F1 (Mags: X400). ***8W*.** VEGF graph of expressions for the period, the same letters (d, e and f) represent equalities among the groups (p > 0.05). Same numbers (I, II and III) represent equalities between same experimental groups (p > 0.05) in different periods (4 vs. 8 weeks).

Positive immunoreactivity for vascular endothelial growth factor (VEGF) was observed 4 weeks after nerve injury in all experimental groups (Fig 3). The immunostaining results of the non-injured groups, Control and Exposed were similar (p > 0.05) observed mainly in the endothelial cell region. The LLLT group presented similar staining (p > 0.05) to the non-injured groups, but with diffuse marking between the axons. The F1 protein and LLLT & F1 groups showed similar immunoreactivity among themselves and were significantly higher (p < 0.05) compared to the non-injured groups. The markers in these groups were less dispersed and concentrated at certain points between axons and glial cells. In this period of analysis, Injured group showed the highest immunoreactivity for VEGF (p < 0.05) being similar only to the LLLT&F1 group. In this case, the marking was diffused throughout the cross-sectional area of the sciatic nerve.

Eight weeks after nerve injury, positive immunoreactivity was observed in all experimental groups (Fig 3). The Control, Exposed, Injury and LLLT groups showed similar immunoreactivity (p > 0.05) occurring mainly in the region of endothelial cells and diffusely between axons and glial cells. In this period of analysis, the F1 protein and LLLT&F1 groups were similar to each other and larger than the other experimental groups, the only exception being group I, which was also similar to F1 and LLLT&F1 groups.

Comparison of VEGF expressions in groups with similar experimental methodology at different periods (4 and 8 weeks) revealed that only Exposed, Injury and F1 groups presented significant differences (p <0.001). Groups Exposed and Injury revealed a significant reduction and the F1 protein group showed a significant increase in VEGF expression.

### Ultrastructural morphology– 4 weeks after nerve injury

**Fig 4:**
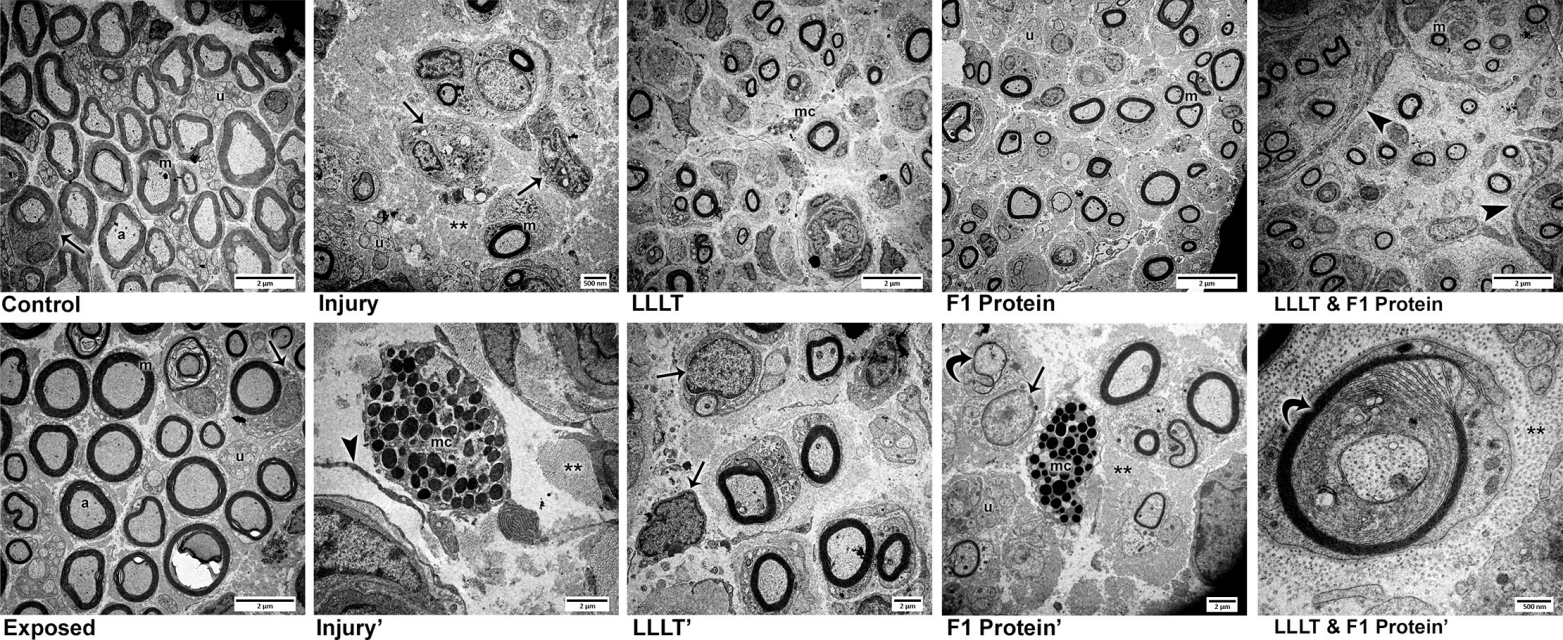
Ultrastructural characteristics (TEM) four weeks after nerve injury. ***Control*.** Control—Clusters of unmyelinated fibers (u), Schwann cells (arrow) involving myelin fibers (m) with their axoplasm (a) containing mitochondria (mag: X2,000). ***Exposed*.** Exposed Nerve—Similar characteristics to the control were observed: presence of myelin (m) and unmyelinated fibers (u) and Schwann cells (arrow) (mag: X2,000). ***Injury*.** Injured Nerve—Large areas of collagen fibers (**), scarce myelin fibers (m), small clusters of unmyelinated fibers (u) and Schwann cells (arrow) were noted (mag: X3,000). ***Injury'*.** In detail, the presence of mast cells (mc) were seen among collagen fibers (**) and near to laminar remains of the Schwann cell cytoplasm (arrow head) (mag: X7,500). ***LLLT*.** Injured Nerve with LLLT—Small clusters of myelinated nerve fibers (m) and the presence of mast cells (mc) were noted (mag: X2,000). ***LLLT'*.** At higher magnification we noticed reactive Schwann cells with large nuclei, one of which was suggestively looking for axons (mag: X5,000). ***F1 Protein*.** Injured Nerve with F1—Small clusters of small caliber myelin fibers (m) and clusters of unmyelinated fibers (u) were observed (mag: X2,000). **F1'.** In detail, extensive areas containing collagen fibers (**) among myelinated and unmyelinated (u) nerve fibers, as well as mast cells (mc) were noted. Schwann cells with bulky nuclei (arrow) close to nerve fibers in suggestive process of remyelination (curved arrow) (mag: X5,000). ***LLLT & F1 Protein*.** Injured Nerve with LLLT and F1—Small clusters of myelin fibers (m) involved by laminar cytoplasmic structures (arrowhead) (mag: X2,000) are noted. ***LLLT & F1 Protein'*.** At higher magnification, a nerve fiber was observed in a suggestive process of remyelination (curved arrow) surrounded by the cytoplasm of a Schwann cell enveloped in collagen fibers (**) of the endoneurium (mag: X25,000).

Ultrastructural analysis using transmission electron microscopy revealed normal features in the non-injured groups, Control (Fig 4 Control) and Exposed (Fig 4 Exposed). Four weeks after of the experimental protocols, the presence of myelin nerve fibers of various diameters in these groups was observed interspersed with clusters of smaller diameter unmyelinated nerve fibers throughout the cross-sectional area of the sciatic nerve. In the axoplasm region of the nerve fibers, there electron-dense circular structures were observed, representing mitochondria of reduced diameter. In addition to the nerve fibers, Schwann cells were also observed with electron-dense nuclei surrounding the myelinated and unmyelinated fibers.

The injured groups presented alterations in the organization of the nervous elements in comparison to the Control and Exposed groups. In general, a reduction in the concentration of myelinic and unmyelinated fibers was noticed, accompanied by a considerable increase of the area occupied by collagen fibers in the endoneurium. Injury group (Fig 4 Injury) had a few scattered myelinated nerve fibers and small clusters of unmyelinated axon and Schwann cells with areas of nerve fiber degeneration. In more detail, mast cells were observed between these nerve fibers and cytoplasmic remnants of the Schwann cells in laminar form (Fig 4 Injury').

The treated groups, LLLT, F1 and LLLT&F1 (Fig 4 LLLT, 4 F1 Protein, 4 LLLT & 4 F1 Protein) presented similar characteristics, such as reduction in the number, density and diameter of nerve fibers compared to Control and Exposed groups, in addition to the greater amount of collagen fibers in the endo and perineurium as observed in Injury group. However, these groups differed from Injury group because they revealed a subtle increase in the density of nerve fibers and showed organization in small clusters. Among them, the characteristics observed in the F1 group suggest the best characteristics, such as slightly calibrous axons and a denser occupation of the cross-sectional area of the sciatic nerve. In more detail, it was possible to notice aspects suggestive of recovery. Schwann cells were noticed in the LLLT group (Fig 4 LLLT') more reactive with more massive nuclei, searching for nerve fibers. In the F1 group (Fig 4 F1 Protein'), small-caliber fibers that appeared to have been remyelinated were observed near a mast cell. In the LLLT&F1 group (Fig 4 LLLT & 4F1 Protein'), a fiber surrounded by a Schwann cell was visible in a process suggestive of remyelination.

### Ultrastructural morphology—8 weeks after nerve injury

**Fig 5:**
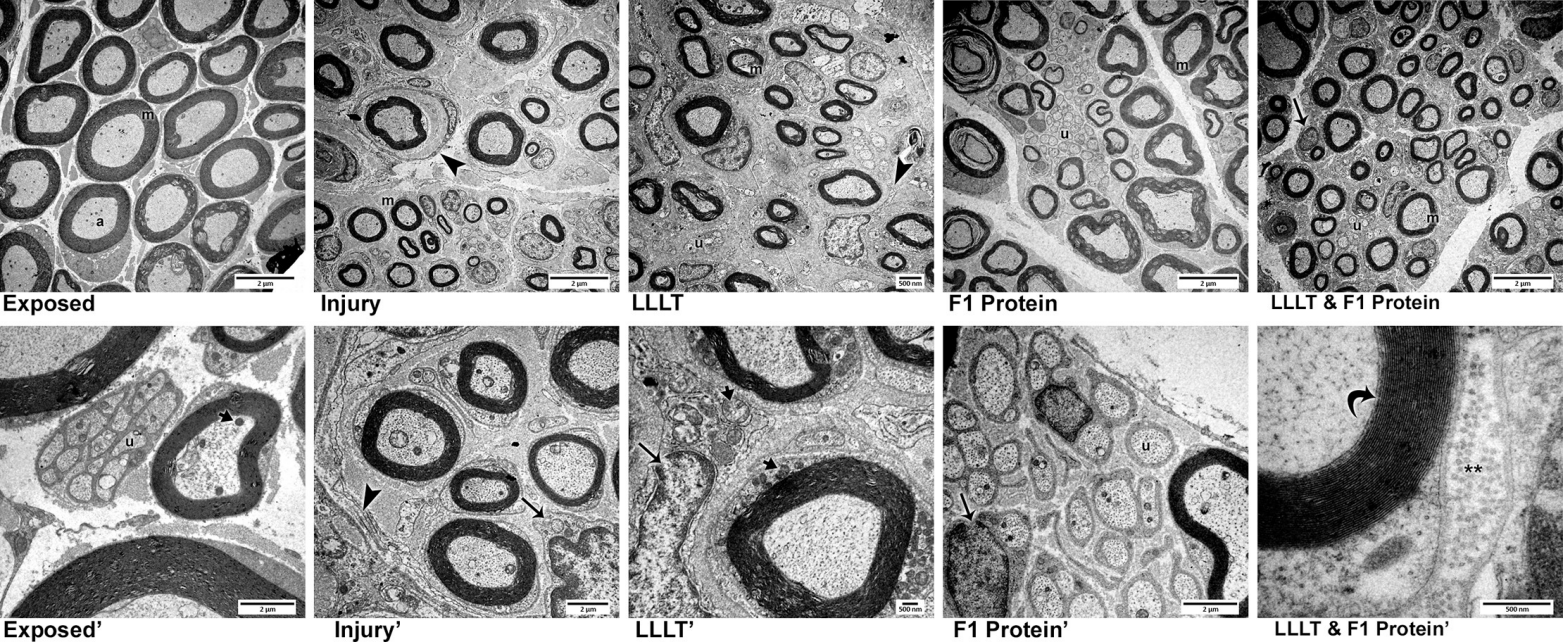
Ultrastructural characteristics (TEM) Eight weeks after nerve injury. *Exposed*. Exposed Nerve—Normal characteristics were observed with the presence of large caliber myelin fibers (m) and the axoplasm (a) region containing mitochondria (mag: X2,000). ***Exposed'*.** At higher magnification, unmyelinated fibers (u) interspersed with myelinated fibers with mitochondria (short arrow) in the axoplasm region are noted (mag: X10,000). ***Injury*.** Injured Nerve—In this period of analysis, small clusters of myelin fibers (m) surrounded by laminar cytoplasmic structures (arrow head) were still observed in this group (mag: X2,000). ***Injury'*.** In detail, a cluster of myelin fibers surrounded by cytoplasmic laminae (arrow head) and close to Schwann cells (arrow) (mag: X7,500). ***LLLT*.** Injured Nerve with LLLT—Similar characteristics to group I, small clusters of myelin fibers (m) and clusters of unmyelinated fibers (u) were noted (mag: X3,000). ***LLLT'*.** At higher magnification, the presence of Schwann cells (arrow) with bulky nuclei containing many mitochondria (short arrow) was observed. Schwan cell cytoplasm involving myelin fiber also revealed mitochondria (short arrow) (mag: X12,000). ***F1 Protein*.** Injured Nerve with F1—Higher densities of myelin (m) and unmyelinated fibers (u) were observed, which were no longer seen to be organized into clusters (mag: X2,000). ***F1 Protein'*.** In detail, unmyelinated fibers (u) surrounded by cytoplasm of Schwann cells were observed, as well as the presence of Schwann cells with bulky nuclei (arrow) (mag: X10,000). ***LLLT & F1 Protein*.** Injured Nerve with LLLT and F1—Characteristics were similar to F1 group, with a dense presence of myelin (m) and unmyelinated fibers (u) and Schwann cells (arrow), without the appearance of clusters (mag: X12,000). ***LLLT & F1 Protein'*.** At higher magnification, lamellae of the myelin sheath (curved arrow) surrounded by the Schwann cell cytoplasm and endoneurial collagen fibers (**) were observed. (mag: X50,000).

Eight weeks after nerve injury, the exposed nerve (Fig 5 Exposed) presented normal ultrastructural characteristics with myelin nerve fibers of various diameters and clusters of unmyelinated fibers throughout the cross-sectional area of the sciatic nerve. At higher magnification, it was possible to observe mitochondria in the axoplasm of myelinated and unmyelinated nerve fibers (Fig 5 Exposed').

The groups submitted to nerve damage (Injury, LLLT, F1 and LLLT&F1) presented better conditions compared to the previous period of analysis, with larger, more densely organized nerve fibers.

Groups Injury (Fig 5 Injury) and LLLT (Fig 5 LLLT) showed similar features maintaining the small clusters of myelinated axons surrounded by laminar cytoplasm of glial cells. At higher magnification (Fig 5 Injury' and 5 LLLT'), myelin fibers of various diameters, clusters of unmyelinated axons and Schwann cells with nuclear electron contents were observed, as well as mitochondria in the axoplasm region of the nerve fibers.

The groups F1 (Fig 5 F1 Protein) and LLLT&F1 (Fig 5 LLLT& 5 F1 Protein) showed similar characteristics during this period, with a higher density of myelinated and unmyelinated axons, resembling the non-injured groups, yet with smaller nerve fiber diameter. The small clusters of myelinated axons were not noted in these groups. At higher magnification, many unmyelinated fibers close to myelin fibers and Schwann glial cells were observed in the F1 group (Fig 5 F1 Protein'). In the LLLT&F1 group (Fig 5 LLLT & 5 F1 Protein') the concentric myelin sheath lamellae were observed, contained in the cytoplasm of the Schwann cells and collagen fibers of the endoneurium region. During this period of analysis, no inflammatory cells and few areas of degeneration were observed in all groups submitted to nerve damage.

## Discussion

The present study analyzed the expressions of the NGF and VEGF growth factors and the ultrastructural morphology of the sciatic nerve after a crush injury and subsequent treatment with LLLT and F1 protein, in order to improve the understanding of the action of these agents and establish a relationship with our previous morphometric studies [21, 22, 24]. Thus, in general, the exposed group (E) showed no significant differences in relation to the control group in both analyzed periods. The injured groups (Injury, LLLT and LLLT&F1) presented higher NGF expressions four weeks after of injury associated with a reduction in organization, density and size of nerve fibers. In this period among the injured groups, only the F1 group had low NGF expression and better morphologic characteristics, with more densely organized nerve fibers. In the 4th week, VEGF expression was significantly higher in groups Injury, F1 and LLLT&F1. Eight weeks after of injury, NGF expression in the injured groups decreased; the morphological characteristics were more similar to the non-injured groups and VEGF expression remained high only in the groups that received the F1 protein (F1 and LLLT&F1).

Neurotrophic factors can be produced spontaneously, however after an axonotmesis injury it is known that complete nerve regeneration depends on highly favorable trophic conditions for neuronal protein synthesis and the expression of these growth factors [12, 42]. NGF is secreted by Schwann cells following nerve damage and after nearly 3–4 weeks their levels decrease [43].

The results of the present study suggest that NGF expression was associated with nerve injury, since in the injured groups there were significant increases in the immunoreactivity of this factor compared to non-injured groups. In addition, manipulation of the sciatic nerve (Exposed group) was not sufficient to result in this increase in NGF expression. In groups Injury, LLLT and LLLT&F1 more intense immunostainings was noted at fourth week of the nerve injury compared to those observed eight weeks after, thus in agreement with the Chang et al. [43].

The 780 nm LLLT used in previous animal studies revealed protective effect, sustenance of functional activity of the injured nerve, decreases scar tissue formation, decreased degeneration in corresponding motor neurons of the spinal cord, significantly increases axonal growth and myelinization [44]. The irradiation of Schwann cell cultures with LLLT (810 nm, 50mW, 1 and 4 J/cm^2^) stimulated the proliferation of these cells and elevated NGF gene expression [13]. In the present study, the increase in NGF immunoreactivity after nerve injury was observed in animals receiving LLLT irradiation, even when using different physical parameters which were employed by Yazdani et al. [13]. However, this increase was also observed in animals that were injured and did not receive treatment, thus is not possible affirm that the increase in the marking of this factor was only due to the LLLT action.

Some studies report the capacity of NGF to induce and mediate angiogenesis in pathological and physiological conditions due to its action on endothelial cells [45–50]. It has been reported that endothelial cells synthesize NGF [51], and that this factor is capable of mediating the action of VEGF [49, 51]. These studies [49, 51, 52] help to elucidate the positive NGF labeling observed in the blood capillaries from our results, showing that this factor may aid in the recovery of vascularization beyond the nerve tissue itself.

The increase in vascular permeability and associated angiogenesis are crucial events for tissue repair, which allows a variety of cytokines and growth factors to reach the injured tissue. Angiogenesis helps to supply tissues with a wide variety of nutrients and release of metabolites. The F1 protein has demonstrated healing properties and high angiogenic activity [53]. The crush-type injury of a peripheral nerve induces pathological modifications of the nervous capillaries, decreasing blood flow and oxygen tension, resulting in ischemia and hypoxia [54].

VEGF is a cytokine that acts on the formation of new capillaries from pre-existing vessels [55]. Angiogenesis repairs ischemia, assisting in nerve regeneration; VEGF and VEGFR2 receptors are major angiogenic modulators which play essential roles in the regulation of vascular neoformation in the initial post-ischemic phases [42]. VEGF can exert non-angiogenic effects on several cell types including nerve cells [56]. It has already been reported that the impregnated VEGF in isografts used for the treatment of nerve grafts increases the number of axons [57].

In the present study, the VEGFs show positive immunoreactivity in all groups regardless of injury and/or treatment. This factor may be stimulated by the damage of the nervous tissue [42, 55]. Our results revealed a significant increase in the expression of this factor at 4 weeks after nerve injury in groups Injury, F1 and LLLT&F1. Eight weeks after of nerve injury, both groups that received the F1 protein (F1 and LLLT&F1) still showed significantly higher VEGF expressions. Comparison of VEGF expressions in both periods revealed a significant reduction in expression in group I and a significant increase in the F1 group 8 weeks after compared to the previous period. Previous studies [58, 59] reported increased VEGF expression in cutaneous lesions with LLLT irradiation, however our results in LLLT nerve damage did not result in this increase.

The data of present study supports the quantitative analysis of blood vessels in our previous study [21] in which, 8 weeks after the nerve injury, increases in capillary density were observed in groups F1 and LLLT&F1, from animals that received the F1 protein. Among the actions of the F1 protein is the recognized capacity of neoformation of blood vessels [18, 19, 53, 60], which may be related to increased VEGF expression in both periods analyzed.

The results of ultrastructural morphology corroborate with the immunohistochemical results and elucidate the changes of each experimental group. The presence of small clusters of myelinated nerve fibers is the morphological characteristic observed in the recovery of the nerve crush-type lesion (axonotmesis) [61–65]. The presence of mast cells, which are cells associated with allergic and non-allergic inflammatory reactions [66, 67] in nerve samples from F1 group animals could raise the suspected allergic reactions associated with protein extracted from the latex. Notwithstanding, the same cells were also observed in injured animals without the application of the protein. This finding suggests that the presence of mast cells would be more associated with their function in immune and inflammatory events. In addition, the ultrastructural analysis supports the characteristics suggestive of recovery in the injured and treated groups with different protocols which would not be observed by conventional microscopy, such as remyelination and reactivity of the Schwann cells.

The molecular mechanisms associated with F1 protein are still poorly understood. Previous studies associate the effect of the F1 protein with immune cells, which can regulate the expression of inflammatory cytokines [68–70]. F1 administration was able to reduce levels of interleukin-1β (IL-1β), interleukin-6 (IL-6) and tumor necrosis factor-α (TNF-α), and increase levels of interleukin-10 (IL-10) and interleukin-4 (IL-4) which in general contribute to an anti-inflammatory effect [68–70]. Our results suggest that F1 protein may be associated with the regulation of growth factors, such as NGF and VEGF. Previous studies have demonstrated the association of NGF expression with IL-1β and TNF-α [71] and VEGF with IL-6 [72]. However, the cause-effect relationship between these growth factors and the already described molecular mechanisms of F1 protein is unclear.

Our results showed a downregulation of NGF in the F1 treated animals mainly after 4 weeks, and also a downregulation of VEGF in the LLLT groups in both evaluated periods. This downregulation of NGF associated with morphometric and ultrastructural results of our previous studies [21, 22] added to the results observed in TEM, allows us to suggest an acceleration of axonal regeneration when applied to F1 protein and thus resulting in a decrease in expression of this factor in both periods analyzed. The low expression of VEGF in LLLT treated nerve lesions is incongruent with other models of tissue injury [58, 59], suggesting that in each type of tissue this treatment modality can regulate this growth factor differently, and also considering differences in the physical parameters of the irradiations.

Among the limitations of the present study is the lack of comparisons between the behaviors of NGF and VEGF against the application of F1 protein associated or not to LLLT, since no previous studies evaluating theses growth factors expression were found. In addition, methods could have been used to quantify the expression of the most accurate growth factors. We justify the non-use of these methods because this is a preliminary morphological study that analyzed the effect of these treatments on nerve injury. Our research group is working to implement more methods of analysis to improve the understanding about the aspects of nerve regeneration associated with LLLT and the F1 protein.

## Conclusions

The treatments protocols applied in present study resulted in improvements in nerve fiber regeneration of the sciatic nerve injured. At 4^th^ week post-injury the F1 group showed lower NGF expression and better ultrastructural characteristics suggesting a recovery acceleration. It was also possible to suggest that the F1 protein and LLLT interaction was negative, since the results observed in the groups receiving LLLT (LLLT and LLLT&F1) were worse in comparison to the F1 group. In addition, regarding the angiogenic capacity of the F1 protein, the maintenance of the expression of VEGF 8 weeks after of injury could be associated with the increase of vessels reported in our previous study [21]. This helps to strengthen the evidence that a main action of this protein would be associated with the capacity of vascular neoformation. On the other hand, these results are only the first step in understanding how these agents act to improve peripheral nerve injuries. Further studies employing different methods of analysis are needed to elucidate the effectiveness of the morphological results presented.

## Supporting information

S1 Table NGF expression. NGF expression data (% area) 4 and 8 weeks after nerve injury.
(DOCX)

S2 Table VEGF expression. VEGF expression data (% area) 4 and 8 weeks after nerve injury.
(DOCX)
