# Supplementary material for: Growth factors expression and ultrastructural morphology after application of low-level laser and natural latex protein on a sciatic nerve crush-type injury
Source: PLoS One. 2019 Jan 9;14(1):e0210211. doi: 10.1371/journal.pone.0210211 (PMC6326513; doi:10.1371/journal.pone.0210211)
Supplement: S1 Table — NGF expression data (% area) 4 and 8 weeks after nerve injury. (DOCX) [file pone.0210211.s001.docx]

| **NGF Expression - 4 weeks (% area)** | | | | | |
| --- | --- | --- | --- | --- | --- |
| **Control** | **Exposed** | **Injury** | **LLLT** | **F1 protein** | **LLLT & F1** |
| 3.288 | 15.965 | 70.519 | 62.84 | 13.685 | 17.381 |
| 9.995 | 3.753 | 64.651 | 68.559 | 13.799 | 12.353 |
| 5.519 | 4.875 | 67.049 | 65.706 | 17.605 | 31.002 |
| 1.545 | 0.46 | 63.619 | 66.422 | 17.314 | 36.354 |
| 6.101 | 0.91 | 51.302 | 64.632 | 17.302 | 15.714 |
| 6.058 | 2.787 | 52.716 | 66.693 | 16.122 | 11.066 |
| 2.06 | 3.941 | 65.841 | 62.169 | 16.907 | 22.14 |
| 5.537 | 0.535 | 55.655 | 52.646 | 19.059 | 26.229 |
| 4.361 | 0.382 | 40.881 | 63.798 | 16.625 | 15.047 |
|  | 11.689 | 41.088 | 56.544 | 19.641 | 14.111 |
|  | 11.574 | 73.789 | 68.48 | 15.67 | 10.852 |
|  | 10.069 | 65.748 | 65.453 | 21.756 | 44.74 |
|  | 20.321 | 69.701 | 71.776 | 20.837 | 61.19 |
|  | 26.264 | 58.63 | 72.492 | 17.187 | 78.785 |
|  | 16.698 | 73.592 | 74.799 | 13.081 | 79.311 |
|  | 20.64 | 72.131 | 71.423 | 21.688 | 76.805 |
|  | 28.165 | 72.941 | 69.517 | 21.109 | 77.579 |
|  |  | 71.653 | 61.907 | 20.344 | 66.74 |
|  |  | 77.328 | 56.945 | 19.647 | 48.981 |
|  |  | 80.036 | 54.199 | 15.152 | 43.552 |
|  |  | 77.989 | 59.101 | 30.469 | 46.807 |
|  |  | 66.848 |  | 17.302 | 73.2 |
|  |  | 59.13 |  | 12.917 | 75.573 |
|  |  | 68.938 |  | 17.454 | 74.066 |
|  |  | 73.213 |  | 32.135 | 77.327 |
|  |  | 73.431 |  | 52.557 | 69.418 |
|  |  | 83.186 |  | 38.556 | 65.157 |
|  |  | 80.79 |  | 42.058 | 48.515 |
|  |  | 72.796 |  | 27.19 | 59.399 |
|  |  | 70.955 |  | 26.174 | 68.242 |
|  |  | 79.599 |  | 16.74 | 76.389 |
|  |  | 88.565 |  | 24.574 | 63.633 |
|  |  | 86.466 |  | 15.439 | 70.28 |
|  |  | 86.884 |  | 8.321 | 70.936 |
|  |  | 85.821 |  | 3.242 | 65.505 |
|  |  | 67.413 |  | 8.004 | 68.298 |
|  |  | 63.8 |  | 26.009 | 69.686 |
|  |  | 67.924 |  | 13.5 | 70.883 |
|  |  | 84.607 |  | 7.049 |  |
|  |  | 76.915 |  | 3.742 |  |
|  |  | 65.282 |  |  |  |
|  |  | 29.004 |  |  |  |

| **NGF Expression - 8 weeks (% area)** | | | | | |
| --- | --- | --- | --- | --- | --- |
| **Control** | **Exposed** | **Injury** | **LLLT** | **F1 protein** | **LLLT & F1** |
| 34.6 | 25.809 | 53.921 | 59.218 | 26.524 | 16.199 |
| 33.51 | 23.542 | 46.321 | 52.519 | 14.721 | 17.129 |
| 23.77 | 18.055 | 33.777 | 56.834 | 18.969 | 18.288 |
| 29.27 | 19.362 | 54.066 | 56.211 | 19.539 | 24.711 |
| 19.75 | 16.257 | 53.325 | 51.588 | 20.793 | 18.798 |
| 9.69 | 19.689 | 55.606 | 53.473 | 19.213 | 20.66 |
| 19.17 | 20.349 | 58.437 | 50.076 | 20.344 | 26.753 |
| 28.46 | 18.708 | 53.32 | 53.264 | 11.661 | 31.156 |
| 13.9 | 17.014 | 54.554 | 52.176 | 19.224 | 29.946 |
| 15.62 | 11.994 | 53.895 | 60.721 | 23.657 | 19.838 |
| 6 | 19.527 | 52.443 | 58.084 | 48.717 | 18.58 |
| 13.15 | 19.658 | 56.562 | 57.188 | 19.119 | 22.738 |
|  | 18.103 | 55.408 | 61.419 | 13.079 | 17.558 |
|  | 54.762 |  | 55.961 | 40.879 | 10.973 |
|  | 37.772 |  | 33.454 | 38.022 | 12.366 |
|  | 38.609 |  | 32.397 | 27.23 | 14.889 |
|  | 35.526 |  | 31.179 | 30.567 | 14.691 |
|  | 37.643 |  | 32.155 | 26.86 | 26.075 |
|  | 39.501 |  | 33 | 30.847 | 28.042 |
|  | 47.869 |  | 28.461 | 36.505 | 13.437 |
|  | 34.83 |  | 28.457 | 19.24 | 15.213 |
|  | 30.967 |  | 29.271 | 28.415 | 22.125 |
|  | 29.15 |  | 32.692 | 31.534 | 21.277 |
|  | 38.367 |  | 37.347 | 28.426 | 21.957 |
|  |  |  | 36.339 | 30.852 | 23.204 |
|  |  |  |  | 33.852 | 17.248 |
|  |  |  |  | 27.643 | 24.501 |
