# Supplementary material for: Growth factors expression and ultrastructural morphology after application of low-level laser and natural latex protein on a sciatic nerve crush-type injury
Source: PLoS One. 2019 Jan 9;14(1):e0210211. doi: 10.1371/journal.pone.0210211 (PMC6326513; doi:10.1371/journal.pone.0210211)
Supplement: S2 Table — VEGF expression data (% area) 4 and 8 weeks after nerve injury. (DOCX) [file pone.0210211.s002.docx]

| **VEGF Expression - 4 weeks (% area)** | | | | | |
| --- | --- | --- | --- | --- | --- |
| **Control** | **Exposed** | **Injury** | **LLLT** | **F1 protein** | **LLLT & F1** |
| 4.122 | 10.441 | 24.775 | 14.409 | 13.269 | 15.74 |
| 2.397 | 12.213 | 17.095 | 11.159 | 11.01 | 17.247 |
| 21.524 | 21.426 | 15.241 | 6.484 | 13.469 | 35.246 |
| 19.655 | 9.854 | 12.681 | 10.821 | 7.57 | 32.349 |
| 3.063 | 11.007 | 15.491 | 10.031 | 6.873 | 17.685 |
| 2.724 | 17.641 | 22.543 | 10.709 | 3.613 | 16.003 |
| 8.341 | 8.042 | 23.782 | 14.602 | 5.4 | 9.999 |
| 5.882 | 6.191 | 15.656 | 16.522 | 6.821 | 19.733 |
| 12.939 | 3.654 | 14.848 | 20.908 | 7.12 | 33.457 |
| 21.693 | 10.721 | 39.152 | 25.863 | 7.669 | 33.477 |
| 15.8 | 22.355 | 39.615 | 27.894 | 28.845 | 19.174 |
| 15.319 | 28.253 | 30.117 | 26.73 | 38.617 | 18.949 |
| 0.318 | 33.197 | 36.567 | 11.183 | 36.045 | 12.463 |
| 3.066 | 23.636 | 34.056 | 10.695 | 52.806 | 21.182 |
| 11.734 | 10.87 | 30.122 | 10.539 | 36.103 | 16.272 |
| 26.943 | 3.798 | 26.329 | 11.807 | 31.228 | 14.457 |
| 21.446 | 7.863 | 25.808 | 16.117 | 42.913 | 25.421 |
| 5.518 | 3.007 | 38.075 | 20.911 | 32.738 | 26.217 |
| 0.294 | 3.294 | 38.947 | 18.762 | 22.576 | 20.918 |
| 0.634 | 2.618 | 28.555 | 16.693 | 24.303 | 14.151 |
| 1.952 | 3.086 | 32.594 | 11.349 | 20.51 | 23.279 |
| 4.606 | 3.02 | 75.54 | 12.817 | 19.096 | 37.543 |
| 20.092 | 4.326 | 50.237 | 13.716 | 19.875 | 35.943 |
| 6.486 | 5.074 | 21.008 | 14.324 | 25.854 | 26.181 |
| 1.389 | 6.473 | 34.978 | 11.763 | 28.055 | 32.709 |
| 0.777 | 4.558 | 36.858 | 13.513 | 26.74 | 34.581 |
| 13.534 | 4.198 | 30.017 | 3.203 | 23.329 | 26.089 |
| 8.347 | 4.601 | 18.73 | 1.902 | 25.267 | 32.519 |
| 8.818 | 7.315 | 43.689 | 1.022 | 17.571 | 33.737 |
| 12.802 | 8.967 | 61.295 | 1.405 | 17.715 | 22.426 |
| 2.186 | 1.929 | 55.51 | 2.017 | 17.441 | 12.916 |
| 2.651 | 1.863 | 41.981 | 1.529 | 21.975 | 17.412 |
| 2.809 | 2.576 | 24.065 | 2.447 | 15.477 | 19.681 |
| 2.835 | 6.219 | 29.052 | 2.059 | 10.988 | 16.229 |
| 4.542 | 6.729 | 23.181 | 8.042 | 8.335 | 16.424 |
| 7.917 | 12.823 | 25.54 | 1.296 | 10.944 | 15.53 |
| 35.447 | 11.669 | 27.17 | 2.511 | 11.91 | 10.489 |
| 35.921 | 12.914 | 59.754 |  | 16.624 | 6.38 |
| 8.489 | 10.746 |  |  | 17.416 | 13.897 |
| 7.468 | 2.087 |  |  |  | 15.288 |
| 4.613 | 1.743 |  |  |  |  |
| 5.048 | 1.522 |  |  |  |  |
|  | 1.554 |  |  |  |  |
|  | 2.415 |  |  |  |  |
|  | 2.763 |  |  |  |  |
|  | 7.035 |  |  |  |  |
| **VEGF Expression - 8 weeks (% area)** | | | | | |
| **Control** | **Exposed** | **Injury** | **LLLT** | **F1 protein** | **LLLT & F1** |
| 0.288 | 1.753 | 13.483 | 8.308 | 35.465 | 13.65 |
| 1.915 | 1.882 | 14.872 | 7.283 | 30.309 | 14.179 |
| 11.957 | 8.078 | 14.152 | 10.302 | 31.42 | 47.219 |
| 31.162 | 4.827 | 8.261 | 8.534 | 27.704 | 24.997 |
| 23.548 | 2.516 | 19.689 | 6.658 | 29.967 | 29.914 |
| 7.452 | 1.897 | 18.96 | 7.857 | 45.825 | 50.197 |
| 2.545 | 1.795 | 36.163 | 15.996 | 40.172 | 14.66 |
| 0.163 | 1.114 | 14.423 | 9.325 | 37.951 | 16.011 |
| 0.333 | 1.878 | 8.443 | 9.683 | 44.99 | 19.714 |
| 2.077 | 2.401 |  | 9.555 | 35.163 | 17.807 |
| 10.027 | 1.722 |  | 10.474 | 34.987 | 18.524 |
| 25.611 | 2.119 |  | 9.514 | 33.224 | 16.867 |
| 20.063 |  |  | 8.71 |  | 17.684 |
| 1.348 |  |  |  |  | 20.85 |
| 1.076 |  |  |  |  | 20.595 |
| 0.64 |  |  |  |  |  |
